# Assessment and Comparison of 3D Conformal Radiotherapy With Volumetric Modulated Arc Therapy for Preserving Remaining Brain Volume in High-Grade Gliomas

**DOI:** 10.7759/cureus.89335

**Published:** 2025-08-04

**Authors:** Harikesh B Singh, Nilesh Mani, Pritanjali Singh, Anil Jaiswal, Samyak Chauhan, Minakshi Mishra

**Affiliations:** 1 Radiation Oncology, All India Institute of Medical Sciences, Patna, IND; 2 Medical Physics, All India Institute of Medical Sciences, Patna, IND

**Keywords:** conformity index, dosimetric comparison, high-grade glioma, homogeneity index, remaining brain volume, three-dimensional conformal radiotherapy, volumetric modulated arc therapy

## Abstract

Introduction

Tri-modality therapy, consisting of maximal safe resection, concurrent chemo-radiation, and adjuvant chemotherapy, is the standard approach for the treatment of high-grade glioma (HGGs). Intensity modulated radiotherapy (IMRT) and volumetric modulated arc therapy (VMAT) have excellent potential to reduce the doses to organs at risk (OARs) with optimal dose conformity. However, the literature on the dosimetric parameters for remaining brain volume (RBV) and its relative comparison is limited.

Methods

We conducted a retrospective study for which the inclusion criteria were being over the age of 18 years and having a histologically proven post-operative HGG with a Karnofsky performance score (KPS) >60. The prescription dose was similar in both techniques (60 Gray in 30 fractions at 2 Gray per fraction). The primary objective was to evaluate and compare the dosimetric profiles of V5, V10, V20, V30, V40, V50, and V60 of the RBV. The secondary objective was to document and compare the doses received by OARs in three-dimensional conformal radiotherapy (3D-CRT) and VMAT.

Results

The planning data of 24 patients were analyzed. The demographic profile was age (<40, 42%; > 40, 58%), male (58%) and female (42%), KPS (60, 12%; 70, 54%; 80, 33%), histopathological grade III (30%) and IV (70%), and tumor location (frontal 50%, parietal 21%, temporal 17%, and occipital 12%). In 79% of the patients, the tumor crossed midline. The VMAT plan showed superior dose conformity compared with the 3D-CRT plan (p-value 0.001). The low-to-medium dose volumes (V5, V10, V20, and V30) of the RBV were found to be statistically significant, thus favoring the 3D-CRT plan, but the medium-to-high dose volumes (V50 and V60), which were high in the 3D-CRT plan, favored the VMAT plan. However, the whole brain mean dose did not show any significant difference.

Conclusion

The VMAT plan showed superior dose conformity compared with the 3D-CRT plan. However, the RBV among the patients receiving the low doses was greater in the VMAT plan than in the 3D-CRT plan, while the RBV among those receiving high doses was significantly greater in the 3D-CRT plan.

## Introduction

The standard of care for high-grade gliomas (HGGs) is maximal safe resection (MSR) followed by adjuvant concurrent chemo-radiotherapy and then adjuvant chemotherapy [1]. Adjuvant radiotherapy is an integral component of the treatment. Median overall survival (OS) is 14-20 months after optimal multimodality treatment. For the past two decades, the standard technique has been three-dimensional conformal radiotherapy (3D-CRT). 3D-CRT is superior to 2D (two-dimensional) RT because it involves shaping radiation beams to match the contour of the tumor. The limitations of 3D-CRT are that a large area of the brain is exposed to radiation, including critical organs at risk (OARs). The pattern of recurrence is usually local [2-4]. The recently developed intensity modulated radiotherapy (IMRT) and volumetric modulated arc therapy (VMAT) techniques have advantages over 3D-CRT. IMRT involves the use of advanced algorithms to modulate the intensity of multiple radiation beams delivered from various angles to achieve high precision in radiation treatment, but it has limitations with respect to longer treatment times and potentially increasing the complexity of treatment planning. Without raising the integral doses or low dose volume, IMRT improves conformity through meticulous planning and prudent beam selection [5]. VMAT is more advanced because it involves the use of a continuous arc, and the intensity of the radiation is modulated as the machine rotates around the patient. VMAT is superior to IMRT in terms of faster planning and treatment, greater efficiency, and excellent tumor conformity. The normal brain tissue is very sensitive to radiation treatment, and most areas of the brain perform specific functions. The purpose of this study was to compare the dosimetric parameters of 3D-CRT with VMAT in the postoperative setting of high-grade gliomas, including the remaining brain volume (RBV).

## Materials and methods

Participants

The aim of this study was to compare and evaluate treatment plans using the VMAT and 3D-CRT techniques for patients with HGGs. The patients were retrospectively selected from our medical record data system. We recruited a total of 24 patients from December 2023 to December 2024. All of the analyses were conducted after proper approval was received from the institute's research advisory committee and ethical committee (IEC No. Ref. No. AIIMS/Pat/IEC/2023/1179). The inclusion criteria for the study were a) 18-70 years of age, b) histopathologically proven post-operative HGG (anaplastic astrocytoma, anaplastic oligodendroglioma, and glioblastoma multiforme), and c) Karnofsky performance score (KPS) ≥70. Patients with a history of multicentric disease or prior radiation were excluded from this study.

Contrast computed tomographic simulation and target volume delineation

Contrast computed tomographic (CT) simulation (GE Health, Germany) was performed in the supine position using a thermoplastic cast for immobilization with a slice thickness of 2.5 mm. All patients postoperatively underwent contrast-enhanced magnetic resonance imaging (CEMRI) consistent with our institutional protocol. CT images of matching patients were fused with T1 contrast, T2 contrast, and fluid-attenuated inversion recovery (FLAIR) sequences of MR images in the digital imaging and communication in medicine (DICOM) format through the use of auto fusion software (deformable registration). Target volume delineation was determined according to the current protocol of the ESTRO-EANO guidelines [6]. Gross tumor volume (GTV) was drawn based on the T1-weighted contrast-enhancing tumor and/or resection cavity, along with any residual contrast-enhancing tumor present using the MR sequence. Clinical target volume (CTV) delineation was performed with an isotropic margin of 1.5 cm around the GTV while respecting anatomical barriers to tumor spread. Non-enhancing areas may represent a component of a glioblastoma (GBM) or HGG as defined in the new World Health Organization classification. In such cases, high T2 or FLAIR signal intensity lesions were incorporated into the CTV. A 5 mm isotropic margin was applied over the CTV to generate the planning target volume (PTV) consistent with our institutional protocol. The OARs, including the eyes, optic nerve, optic chiasma, and brainstem, were contoured. We assumed that brain volume other than the target volume is also an OAR. Accordingly, the whole brain was contoured, including the target volume, and the further RBV was calculated by subtracting the PTV with a further margin of 5 mm. The 3D-CRT and VMAT groups' target and OAR volumes for the patients under investigation were identical, thus reducing the inter-observer variability in volume delineation.

Treatment planning, dose prescription, and constraints

In this study, 3D-CRT and VMAT plans were generated using our planning station for the same target volume. For 3D-CRT, the collapse cone algorithm (MONACO, version 6.00.01) was used, and the Monte Carlo algorithm (MONACO, version 6.00.01) was used for VMAT. The dose prescription was 60 Gray in 30 fractions, 2 Gray per fraction daily, and five fractions per week for both 3D-CRT and VMAT. For plan acceptance, taking into account the dose limit for OARs, both 3D-CRT and VMAT plans were developed to achieve ≥95% coverage of the PTV by 95% of the prescribed dose. Additionally, hot spots (>110% of the prescription doses) were limited to <20% of the PTV and to <2% of the outside. As low as reasonably achievable was followed for the RBV [7-9]. The dose constraints for the OARs are depicted in Table 1.

**Table 1:** Organs at risk, dose constraints

Organs at risk	Dose constraints
Brainstem, maximum dose	<54 Gray
Optic nerve, maximum dose	<54 Gray
Optic chiasma, maximum dose	<54 Gray
Lens maximum, dose	<8 Gray
Eyes, mean dose	<25 Gray

Dosimetric parameter recording and reporting

The 3D-CRT and VMAT plans were compared for every patient individually, slice by slice, and by dose volume histogram (DVH). The parameters were PTV coverage, dose to OARs, the conformity index (CI), the homogeneity index (HI), and V5, V10, V20, V30, V40, V50, and V60 of the RBV. The formulation used for the CI was VRI/TV, where the volume of the reference isodose (VRI) is the volume covered by 95% of the isodose lines, and TV is the target volume [10]. The CI should be equal to 1; a CI <1 indicates that the target volume was not adequately irradiated, and a CI >1 indicates that the irradiated volume was greater than the target volume and healthy tissue was affected. The formulation used for the HI was D5/D95, where D5 is the minimum dose in 5% of the PTV, indicating the "maximum dose", and D95 is the minimum dose in 95% of the PTV, indicating the "minimum dose". Low index values (close to 1) indicate that the dose is relatively homogeneous [11].

Statistical analysis

We used SPSS software (version 20.0; IBM Inc., Armonk, New York) to conduct the statistical analyses. We conducted a descriptive analysis of the dosimetric and demographic data. We obtained a summary of the statistics, including mean, median, range, and standard deviation (SD). The paired t-test performed for all of the analyses yielded p-values of <0.05, indicating that the results were statistically significant.

## Results

We recruited a total of 24 patients for this study, with a median age of 44 years (range 17-73 years), 42% of whom were female and 58% were male. Seventy percent of the patients had grade IV tumors, 50% of which were in frontal locations. Table 2 presents the patients' demographic characteristics.

**Table 2:** Patients' demographic characteristics

Patient characteristics	N=24 (%)
Age group (years)	
<40	10 (42%)
>40	14 (58%)
Gender (%)	
Male	14 (58%)
Female	10 (42%)
Karnofsky performance score (%)	
60	3 (12%)
70	13 (54%)
80	8 (33%)
World Health Organization grade (%)	
III	7 (30%)
IV	17 (70%)
Location (%)	
Frontal	12 (50%)
Parietal	5 (21%)
Temporal	4 (17%)
Occipital	3 (12%)

Planning target volume dosimetry

**Figure 1:**
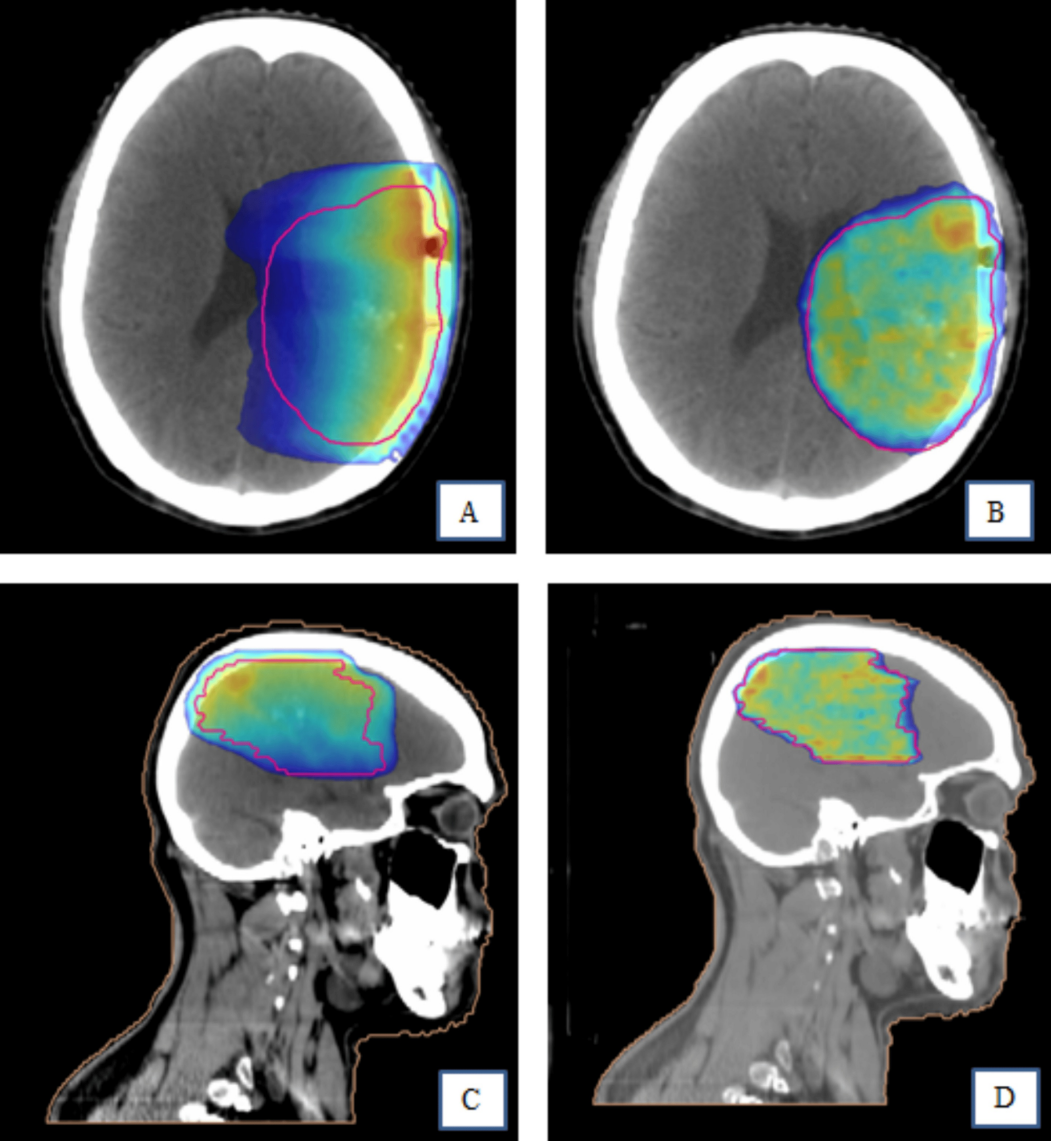
Ninety-five percent isodose Panels A and B show the 95% isodose for three-dimensional conformal radiotherapy and volumetric modulated arc therapy in axial view. Panels C and D show the 95% isodose for three-dimensional conformal radiotherapy and volumetric modulated arc therapy in the sagittal view. Pink - planning target volume

The PTV coverage (D95%) was adequate for all of the plans. Figure 1 shows the 95% isodoses, displayed in axial view and sagittal view.

**Figure 2:**
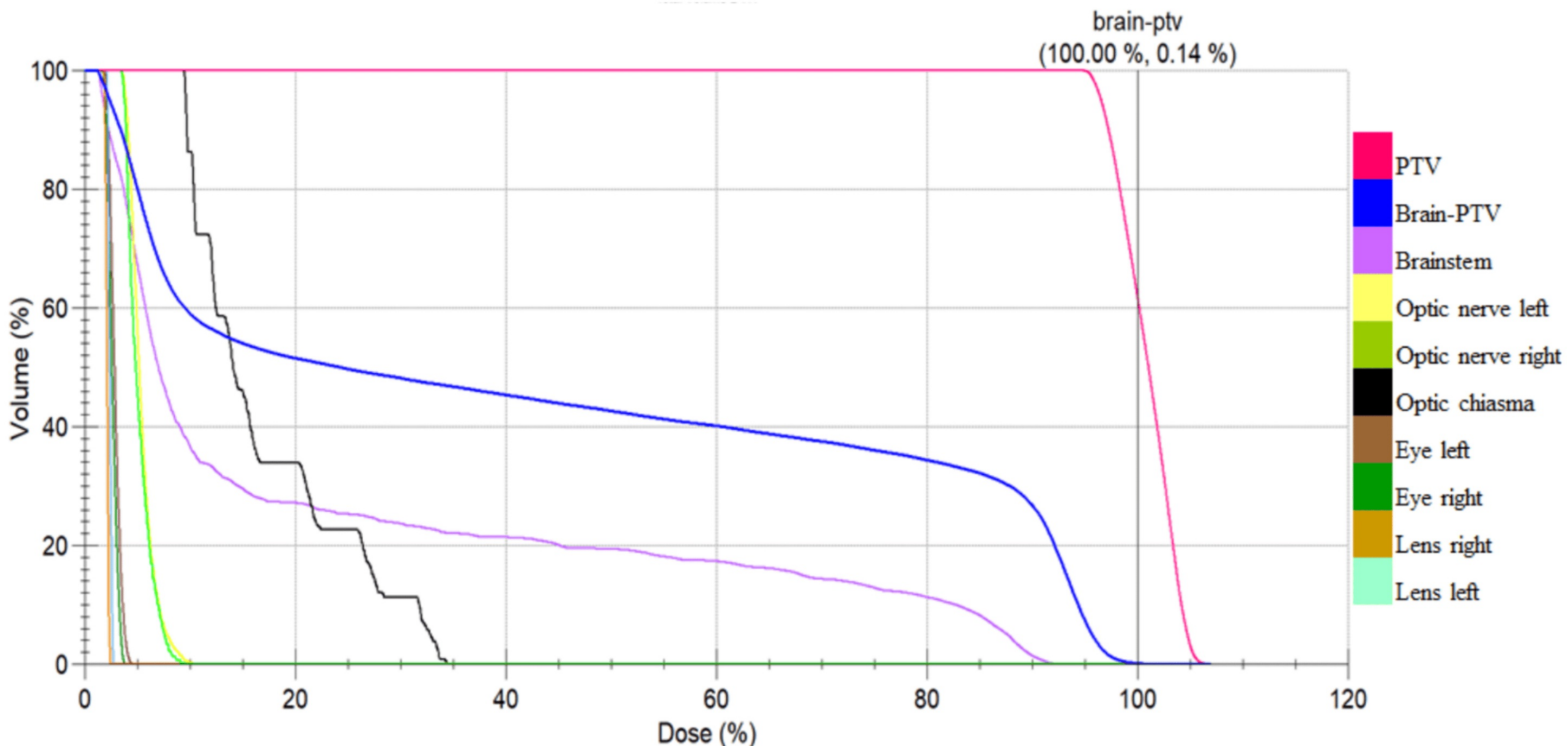
Dose volume histogram analysis for three-dimensional conformal radiotherapy 0.14% of the remaining brain volume received 100% of the prescribed dose (60 Gray). The dose volume histogram of the remaining brain volume (brain-planning target volume) and brainstem becomes less steep, with 20% to 80% of the prescribed doses for the given doses of volume being high PTV - planning target volume

**Figure 3:**
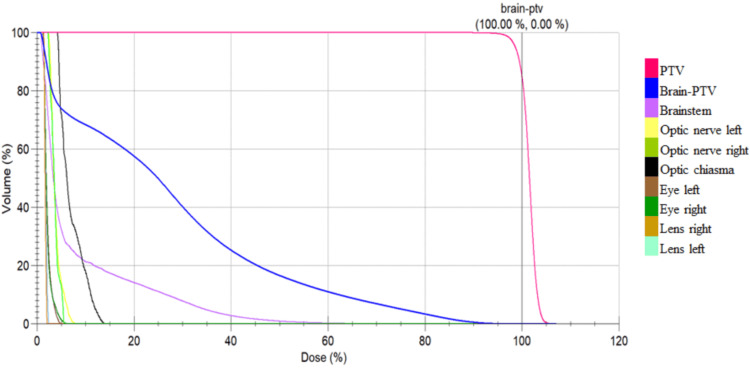
Dose volume histogram analysis for volumetric modulated arc therapy The volume histograms of remaining brain function (brain-planning target volume) and other organs at risk are steeper than in three-dimensional conformal radiotherapy PTV - planning target volume

Conformity was superior with VMAT in all of the patients. The mean PTV coverage at 95% of the prescribed dose was 96.06% for 3D-CRT and 98.22% for VMAT. However, the HI was not found to be significant (mean 1.06±0.03 compared with 1.06±0.12). Figures 2 and 3 show the dose volume histograms for both 3D-CRT and VMAT.

OAR dosimetry

The 3D-CRT technique resulted in a higher maximum dose to the OARs using both techniques. Table 3 presents an overview of these results.

**Table 3:** Overview of the results A paired t-test (two-tailed) was performed, and the results were considered statistically significant when p-values were ≤ 0.05.

Structure (organs at risk and remaining brain volume)	Three-dimensional conventional radiotherapy (mean±standard deviation)	Volumetric arc radiotherapy (mean±standard deviation)	t score	p-value
V5 (%)	47.88 (±19.09)	75.09 (±19.02)	-7.19	0.008
V10 (%)	38.53 (±16.83)	64.46 (±19.53)	-7.51	0.003
V20 (%)	32.75 (±16.54)	39.86 (±16.64)	-2.58	0.017
V30 (%)	28.96 (±15.92)	22.11(±10.71)	2.51	0.02
V40 (%)	24.83 (±15.88)	10.45 (6.49)	4.54	0.216
V50 (%)	20.07 (±12.05)	3.95 (±3.25)	6.49	0.644
V60 (%)	4.65 (±4.66)	0.71 (±3.25)	3.6	0.002
Whole brain (mean dose, Gy)	27.6 (±9.12)	26.88 (±7.59)	0.55	0.588
Brain stem (maximum dose, Gy)	45.54 (±17.18)	44.99 (±15.16)	0.4	0.691
Optic nerve left (maximum dose, Gy)	25.00 (±22.11)	22.68 (±14.61)	0.66	0.551
Optic nerve right (maximum dose, Gy)	27.50 (±24.87)	25.57 (±18.24)	0.57	0.576
Optic chiasma (maximum dose, Gy)	33.87 (±22.84)	37.35 (±17.57)	-1.27	0.217
Lens left (maximum dose, Gy)	3.92 (±2.97)	4.79 (±2.38)	-1.25	0.224
Lens right (maximum dose, Gy)	3.62 (±2.97)	5.06 (±2.91)	-2.64	0.003
Planning target volume V95 (%)	96.06 (±2.60)	98.22 (±1.41)	-3.9	0.001
Homogeneity Index	1.06 (±0.03)	1.06 (±0.12)	0.09	0.93
Conformity Index	0.95 (±0.02)	0.98 (±0.01)	-4.18	0.001

Despite the increased maximum dose (Dmax) (brainstem and optic structures) in 3D-CRT, these results were not found to be significant in the present study. However, the Dmax for the lens was higher in the VMAT technique and was significant for the right lens.

Remaining brain volume dosimetry

**Figure 4:**
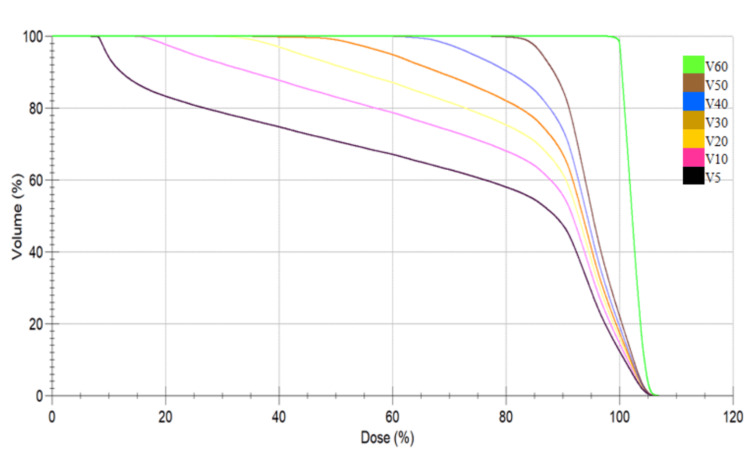
Dose volume histogram analysis of three-dimensional conformal radiotherapy Notes: The dose gradients of V5, V10, V20, V30, V40, and V50 change rapidly after 80% to 100% of the prescribed dose.

**Figure 5:**
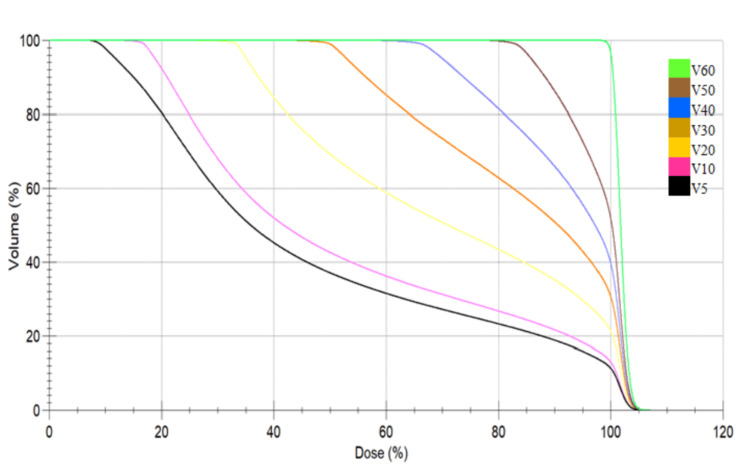
Dose volume histogram analysis of volumetric modulated arc therapy. Notes: The dose gradients of V5 (10%-100% of the prescribed dose) and V10 (15%-100% of the prescribed dose) are steeper than in three-dimensional conformal radiotherapy. The decreases in doses started very early and specifically in low-dose region.

The mean dose of the brain was slightly higher (27.6±9.12 for 3D-CRT compared with 26.88±7.59 for VMAT). Table 3 shows the RBV parameters, including V5, V10, V20, V30, V40, V50, and V60. To enhance clarity, the RBV receiving 5 Gray to 30 Gray was categorized as a low-to-medium dose volume, while the RBV receiving >30 Gray was classified as a high dose volume. The RBV receiving low to medium doses (5 Gray to 30 Gray) was higher with the VMAT technique than with the 3D-CRT technique, and this result was statistically significant. The RBV receiving 60 Gray (mean 4.65±4.66 for 3D-CRT and 0.71±3.25 for VMAT) was higher in the 3D-CRT technique and statistically significant. Figures 4 and 5 show the DVH of the RBV for both 3D-CRT and VMAT.

## Discussion

Adjuvant radiation treatment is the key component in the management of HGG. With the introduction of MRI, linear accelerator technologies, patient immobilization, and advanced algorithms, conformity has improved significantly in terms of sparing normal tissue [12]. In developing countries, where most radiotherapy centers lack advanced technologies or have large numbers of patients and a long waiting list for treatment, 3D conformal treatment remains the standard of care. In recent decades, IMRT and VMAT have become popular because these techniques are simple, allow for rapid planning, and conform to the increased emphasis on sparing normal tissue.

The standard of care for HGGs is the maximal safe resection followed by adjuvant radiotherapy and chemotherapy. Unfortunately, HGGs develop at various sites within the brain, sometimes close to OARs (brainstem, optic nerve, chiasma, etc.). Achieving sufficient coverage of the target volume while preserving OARs is a significant challenge when treating GBM with radiation therapy, as demonstrated by quality assurance for randomized Phase III EORTC 26981/22981 and NCIC CE3 [13]. Beyond the multiple OARs, the brain itself is a vital structure. The RBV is defined as the volume of brain tissue that is spared or unaffected by tumor growth and radiation therapy. The prescription and distribution of the dose are crucial aspects of radiotherapy planning that further depend on the target volume, location, and technique. 3D-CRT involves the use of static beams that conform to the shape of the target volume. However, because of the fixed beam geometry, it is challenging to achieve the optimal distribution of the dose to the target volume with maximal sparing. The effects of late radiation toxicity include neurocognitive impairment, necrosis, memory deficits, and behavioral and personality changes.

The aim of the present study was to provide data on and compare 3D-CRT and VMAT plans and, thereby, determine which technique is superior and identify scenarios in which the optimal strategy and technique provide better dosimetric results. Regarding the OARs, dose constraints to the brainstem and optic structure were achieved. A significant difference was observed in the right lens. The probable explanation for this result is the frontal location of the tumor (approximately 50%) and more scattering in the VMAT plan to achieve optimal target volume coverage. Despite achieving the OAR dose constraints, the dosimetric profile was superior for VMAT. In 3D-CRT, 2.89%, 1.20%, 9.28%, and 7.00% higher mean doses were found for Dmean whole brain, Dmax brainstem, and Dmax optic nerve for the left and right, respectively.

In our study, we achieved optimal target coverage and conformity (PTVV95) with both the 3D-CRT and VMAT plans, but the latter plan achieved statistically significant superiority (98.2% compared with 96.0%), especially when the PTV was close to OARs. Several comparative studies have been conducted over the past few years, and nearly all have suggested that VMAT techniques reduce the doses to OARs while maintaining the optimal target volume coverage. In comparative dosimetric studies, Wagner et al. [14] and Thilman et al. [15] found that IMRT achieved better target coverage, with improvements of 13.5% and 13.1%, respectively, for V95. In the present study, we found a 2.19% increase in conformity with VMAT, consistent with the results of MacDonald et al. [16] and Zach et al. [17]. Table 4 presents a comparison of our results with those in the relevant literature.

**Table 4:** Comparison of dose received by normal structures in various studies.

Studies	Techniques	Brainstem, maximum dose (Gy)	Optic chiasm, maximum dose (Gy)	Optic nerve, maximum dose (Gy)	Lens, maximum dose (Gy)	Whole brain, mean dose (Gy)	Conformity index	Homogeneity index
Shaffer et al., 2010 [18]	Intensity modulated radiotherapy	55.6	52.2	Ipsilateral: 51.9; Contralateral: 41.9	Right lens: 8.7; Left lens: 8.6	22.7	1.16	1.15
	Volumetric arc radiotherapy	55.6	52.5	Ipsilateral: 51.9; Contralateral: 35.2	Right lens: 6.1; Left lens: 6.1	24.7	1.18	1.15
Chan et al., 2003 [19]	Intensity modulated radiotherapy	59	49	24	3.1	32	-	-
	Volumetric arc radiotherapy	58	43	23	2.9	27	-	-
Thibouw et al., 2018 [20]	Three-dimensional conformal radiotherapy	53.2	40	17.25	-	27.8	1.53	-
	Intensity modulated radiotherapy	54.8	52.9	34.2	-	25.7	1.25	-
Ibis et al., 2018 [21]	Three-dimensional conformal radiotherapy	54.6	44	44.2	4.9	32.3	2.3	-
	Volumetric arc radiotherapy	47.2	41.7	Contralateral: 23.9; Ipsilateral: 37.3	Contralateral: 6.2; Ipsilateral: 6.8	23.8	1	-
Our study	Three-dimensional conformal radiotherapy	45.54	33.87	Right optic nerve: 27.5; Left optic nerve: 25	Right lens: 3.62; Left lens: 3.92	27.6	0.95	1.06
	Volumetric arc radiotherapy	44.99	37.35	Right optic nerve: 25.57; Left optic nerve: 22.68	Right lens: 5.06; Left lens: 4.79	26.88	0.98	1.06

MacDonald et al. [16] compared 3D-CRT with an IMRT plan dosimetrically and concluded that IMRT decreased the volumes of the brainstem and optic chiasm receiving more than V45 by 31% and 30.4%, respectively, while the normal brain receiving V18 and V24 decreased by 10% and 14%, respectively. Shaffer et al. [18] showed that lower OAR doses with VMAT achieved optimal PTV coverage at the expense of an increased mean dose to normal brain structures. Chan et al. [19] demonstrated that, compared with 3D-CRT, IMRT provided higher doses (in excess of 10 Gray) to GTV while maintaining the same normal tissue dose constraints. Thibouw et al. [20] compared 3D-CRT and IMRT regarding dosimetric and clinical factors with survival data for GBM patients and concluded that the dose conformity was superior in those treated with IMRT, though the Dmax of the brainstem, optic apparatus, and cochlea was higher. Ibis et al. found that brain-PTV was spared more in IMRT and that the optic chiasm, contralateral optic nerve, and ipsilateral or contralateral cochlea were significantly spared in IMRT and VMAT [21]. The best sparing of the brainstem, pituitary gland, ipsilateral eye, and ipsilateral lacrimal gland was obtained with VMAT. The brain-PTV volume receiving at least 5 Gray was similar in the three plans but lower with 50 Gray in IMRT and VMAT (p<0.001). Although a homogeneous dose distribution was obtained with similar HIs using all three planning techniques, the conformity index was best in VMAT (p<0.001).

According to an analysis by Lorentini et al. [22], in GBM patients treated with both 3D-CRT and IMRT, the latter resulted in better tumor coverage, with a similar dosage to OARs and significantly lower irradiation of healthy brain tissue. In this study, the patients were categorized based on whether there was any overlap between the PTV and dose-limiting normal tissues. We observed no statistically significant variation among the doses received by the PTV, the brainstem, or the optic apparatus when there was no overlap between the two volumes, and the 3D-CRT in the patients was as well-planned as the IMRT. However, we observed differences between the two plans in cases of overlapping volumes, with the IMRT resulting in significantly lower doses to the brainstem and ipsilateral optic apparatus.

 A large volume of healthy brain tissue receives high doses in 3D-CRT, while VMAT allows for more precise dose delivery, thus better sparing the surrounding RBV. We compared V5, V10, V20, and V30 (the low to medium dose volumes) for both 3D-CRT and VMAT and found that the mean doses were high and statistically significant. This finding indicates that 3D-CRT was superior. The V5 Gray volume was 1.5 times greater in VMAT, and the V60 Gray volume was five times greater in 3D-CRT. Interestingly, V60 was 0.71 cc (±3.25) and 4.65 cc (±4.66) in VMAT and 3D-CRT, respectively. Thus, in 3D-CRT, the high dose volume, especially the mean V60, increased. Table 5 presents a comparison of the dosimetric profile of normal healthy brain tissue with that of Lorentini et al. [22].

**Table 5:** Dosimetric comparison of remaining brain volume in a previous study and our study A paired t-test (two-tailed) was performed, and the results were considered statistically significant when p-values were ≤ 0.05. In our study, the t scores were V5 (-7.19), V10 (-7.51), V20 (-2.58), V30 (2.51), V40 (4.54), V50 (6.49), and V60 (3.6).

Dose level	Lorentini et al., 2013 [22]				Our study			
	Intensity modulated radiotherapy	Three-dimensional conformal radiotherapy	Difference	p-value	Volumetric arc radiotherapy	Three-dimensional conformal radiotherapy	Difference	p-value
V5 (%)	74.42	80.63	-6.20	0.04	75.09 (±19.02)	47.88 (±19.09)	+27.21	0.008
V10 (%)	60.67	73.45	-12.78	<0.01	64.46 (±19.53)	38.53 (±16.83)	+25.93	0.003
V20 (%)	36.59	50.87	-14.28	<0.01	39.86 (±16.64)	32.75 (±16.54)	+7.11	0.017
V30 (%)	24.31	32.77	-8.46	<0.01	22.11 (±10.71)	28.96 (±15.92)	-6.85	0.02
V40 (%)	15.86	22.08	-6.22	0.01	10.45 (±6.49)	24.83 (±15.88)	-14.38	0.22
V50 (%)	9.46	15.73	-6.27	0.01	3.95 (±3.25)	20.07 (±12.05)	-16.12	0.64
V60 (%)	2.34	5.93	-3.59	0.05	0.71 (±3.25)	4.65 (±4.66)	-3.94	0.002
Mean dose (Gy)	19.5	23.6	-4.56	<0.001	27.6 (±9.12)	26.88 (±7.59)	+0.72	0.59

The clinical benefit in terms of the survival advantage remains investigational. Huilgol et al. [23] conducted a retrospective study of 46 patients treated with 60 Gray of 3D-CRT and IMRT and found no difference in overall survival. We suggest that VMAT could be advantageous in terms of offering improved clinical benefits in situations in which the PTV is close to OARs. The RBV may be considered as important as OARs. We found the high dose volume to be significantly greater in 3D-CRT, perhaps reflecting late neurological toxicity. A limitation of our study is its retrospective design. A large sample size and long-term clinical data could yield superior results in future studies.

## Conclusions

In the present study, we found that adjuvant radiotherapy utilizing either 3D-CRT or VMAT was satisfactory regarding target coverage and homogeneity. However, VMAT demonstrated greater effectiveness in achieving dose conformity and protecting healthy brain tissue at medium to high doses (V40, V50, and V60). Therefore, VMAT should be considered a suitable treatment option. We conclude that, despite the improved dosimetry, the practical transition to VMAT-based treatment should only be done after careful consideration of the potential consequences.
